# Supplementary material for: Intranasal Delivery of miR133b in a NEO100-Based Formulation Induces a Healing Response in Spinal Cord-Injured Mice
Source: Cells. 2023 Mar 18;12(6):931. doi: 10.3390/cells12060931 (PMC10047048; doi:10.3390/cells12060931)
Supplement: Supplementary file 1 [file cells-12-00931-s001.zip › cells-2147886-supplementary.pdf]

Supplemental data 1:

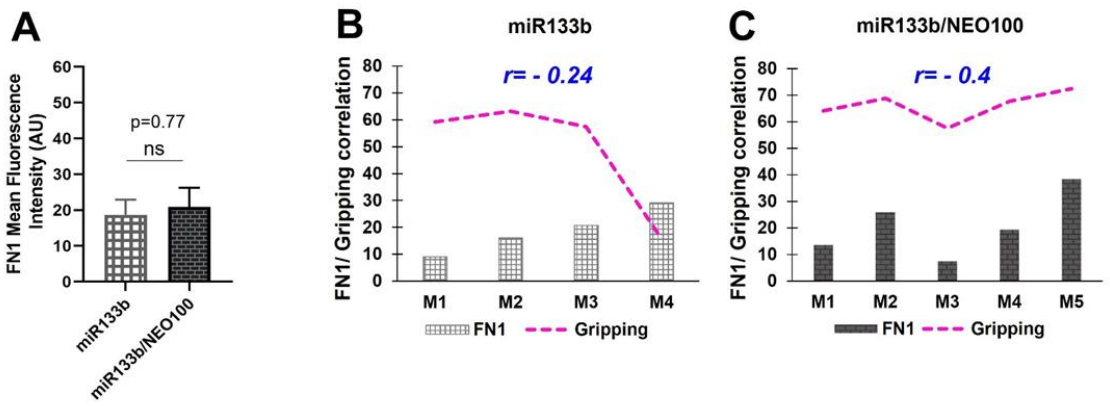

Figure S1. Panel A shows the level of FN1 expression at the lesion site in mice treated with miR133b/Ago2 alone versus miR133b/Ago2 with NEO100 at 56 days post-injury; t-test analysis. Panels B and C show the Pearson correlation coefficient between FN1 expression at the lesion and the forelimb gripping performance with both left and right forepaws at 56 days post-injury; confidence interval 95%. M= FN1 expression per mouse.

Supplemental data 2

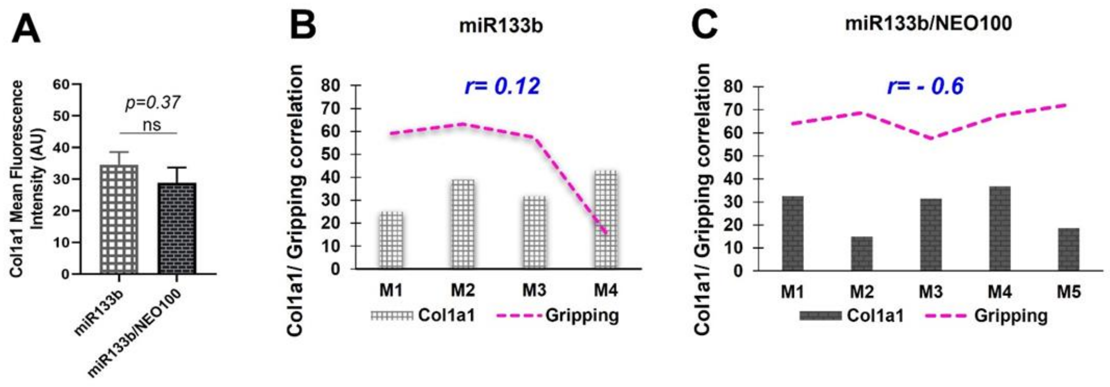

Figure S2. Panel A shows the level of Col1a1 expression at the lesion site in mice treated with miR133b/Ago2 alone versus miR133b/Ago2 with NEO100 at 56 days post-injury; t-test analysis. Panels B and C show the Pearson correlation coefficient between Col1a1 expression at the lesion and the forelimb gripping performance with both left and right forepaws at 56 days post-injury, confidence interval 95%. M= Col1a1 expression per mouse.
